# Differential Prefrontal Hypoactivity in Generalized Anxiety Disorder: The Influence of Nonemotional and Emotional Stimuli in a Go/No‐Go Task

**DOI:** 10.1155/da/5533403

**Published:** 2026-07-30

**Authors:** Chien-An Chen, Yi-Jing Huang, Hsiao-Ting Meng, Pai-Yi Hsu, Yee-Han Keng, Cheng-Yang Lee, I-Ming Chen, Yi-Ting Lin, Yu-Jui Huang, Pao-Huan Chen, Jia-Jin Chen

**Affiliations:** ^1^ Department of Biomedical Engineering, National Cheng Kung University, Tainan, Taiwan, ncku.edu.tw; ^2^ School of Occupational Therapy, National Taiwan University, Taipei, Taiwan, ntu.edu.tw; ^3^ Department of Physical Medicine and Rehabilitation, National Taiwan University Hospital, Taipei, Taiwan, ntuh.gov.tw; ^4^ Lee Cheng-Yang Psychiatric Clinic, Taipei, Taiwan; ^5^ Department of Psychiatry, National Taiwan University Hospital, Taipei, Taiwan, ntuh.gov.tw; ^6^ Department of Psychiatry, Taipei Medical University Hospital, Taipei, Taiwan, tmuh.org.tw

**Keywords:** emotion, fNIRS, generalized anxiety disorder, inhibition, prefrontal cortex

## Abstract

**Background and Objectives:**

Generalized anxiety disorder (GAD) is marked by emotional dysregulation and impaired inhibitory control, often linked to atypical prefrontal cortex (PFC) function. However, how emotional context influences PFC engagement during inhibition remains unclear. This study aimed to examine how emotional versus nonemotional contexts modulate PFC activity in GAD compared to healthy controls (HCs).

**Methods:**

Functional near‐infrared spectroscopy (fNIRS) assessed bilateral PFC activity in 23 individuals with GAD and 42 HC during emotional and nonemotional Go/No‐Go tasks. Two‐way repeated‐measures ANOVAs (group × condition) were conducted per channel with false discovery rate (FDR) correction. Effect sizes are reported as partial eta squared.

**Results:**

A significant group × condition interaction was found for No‐Go accuracy (*p* = 0.032, *η*_p_^2^ = 0.074), with GAD showing a larger decline from nonemotional to emotional conditions (87.70% vs. 47.17%) than HC (79.49% vs. 48.54%). For PFC activity, group × condition interactions reached uncorrected significance in the left dorsolateral PFC (DLPFC) and right dorsomedial PFC (DMPFC) (*p* = 0.013–0.047, *η*_p_^2^ = 0.061–0.094), and the group main effect was uncorrected significant across bilateral dorsomedial and dorsolateral PFC (DLPFC, *p* = 0.005–0.039, *η*_p_^2^ = 0.066–0.130); neither survived FDR correction. Significant condition main effect in the right dorsomedial and right ventromedial PFC (VMPFC, *η*_p_^2^ = 0.120–0.159) were revealed after FDR correction.

**Conclusion:**

Both groups showed PFC deactivation under emotional load, most prominently in the right medial PFC. HC exhibited context‐dependent modulation of the left dorsolateral and right DMPFC, whereas GAD showed a flat, unmodulated response and broadly lower PFC activation, alongside selective impairment of response inhibition. These preliminary findings suggest prefrontal inflexibility as a core neural feature of GAD and underscore the role of left dorsolateral and DMPFC in emotion–cognition integration.

## 1. Introduction

Generalized anxiety disorder (GAD) is a prevalent and often debilitating psychiatric condition characterized by excessive and uncontrollable worry, leading to persistent anxiety and significant impairments in emotional, cognitive, and social functioning [1]. While emotional dysregulation is a hallmark of GAD, growing evidence also highlights co‐occurring deficits in cognitive control, particularly in executive functions such as inhibitory control [2]. These emotional and cognitive impairments are believed to interact dynamically, contributing to the onset and maintenance of anxiety symptoms through a self‐reinforcing cycle [3, 4]. One key mechanism involves difficulties maintaining goal‐directed behavior when confronted with emotionally salient or threatening stimuli, which tend to capture attention and interfere with executive processes such as inhibition and attentional shifting [5].

Mounting neuroimaging evidence suggests that these affective and executive dysfunctions in GAD are closely linked to atypical engagement of the prefrontal cortex (PFC) [6, 7], a key neural substrate for integrating emotion regulation and cognitive control. In particular, the dorsolateral PFC (DLPFC) is involved in maintaining task goals and suppressing distracting stimuli [8], the dorsomedial PFC (DMPFC) supports self‐monitoring and conflict resolution [9], and the ventromedial PFC (VMPFC) modulates emotional responses through its connections with limbic structures [10]. In GAD, disrupted functioning across these PFC subregions has been associated with excessive threat monitoring, impaired inhibitory control, and difficulty flexibly regulating emotional responses. For instance, reduced activity in the DLPFC and DMPFC has been linked to inefficient allocation of executive resources, contributing to inflexible threat processing and persistent worry [11]. Additionally, diminished VMPFC activity has been linked to poor discrimination between threat and safety cues, suggesting impairments in the contextual modulation of fear [12]. Together, these findings point to a broader failure of prefrontal systems to adaptively regulate shifting emotional and cognitive demands in GAD.

Despite these insights into general prefrontal dysregulation, the precise ways in which emotional context modulates prefrontal function during cognitive control in GAD remain incompletely understood. This gap is critical, given that individuals with GAD show both attentional biases toward emotionally salient content and reduced cognitive flexibility [13, 14]. It remains unclear whether their prefrontal dysfunction reflects a general inhibitory deficit or a specific vulnerability to emotional interference. According to Pessoa’s dual competition framework [15], emotionally salient stimuli compete for both perceptual and executive resources. In GAD, this competition may be exacerbated by attentional bias, wherein threat‐related information, whether external or internally generated through worry, preoccupies attention and consumes cognitive resources. This persistent overload may limit the flexible engagement of prefrontal control systems in emotionally demanding contexts. Although attentional bias has been consistently documented in GAD [13], it often co‐occurs with insufficient PFC recruitment, suggesting a mismatch between heightened emotional salience and inadequate top‐down regulation.

To date, few neuroimaging studies have directly examined inhibitory control under emotional load in GAD, and the findings on prefrontal engagement remain mixed. A recent study using an emotional Go/No‐Go task reported no significant differences in PFC activation between GAD and healthy controls (HCs) [16]. However, the absence of a nonemotional condition in that design limits interpretation as it remains unclear whether GAD‐related differences in PFC activity are specific to emotional contexts or reflect a more general inhibitory control deficit. Meanwhile, studies using emotionally distracting stimuli in working memory tasks have revealed both PFC hypo‐ and hyperactivation in GAD, depending on task demands and emotional load [17–20]. These inconsistencies underscore the need for a more direct, within‐subject comparison across emotional and neutral contexts using a task specifically designed to probe inhibitory control.

Thus, to clarify the neural mechanisms underlying inhibitory control in emotional contexts, this study compared prefrontal activation patterns between patients with GAD and HC during emotional and nonemotional Go/No‐Go tasks. Specifically, we aimed to (1) examine whether emotional interference differentially modulates PFC engagement during inhibition across groups and (2) explore how prefrontal activation relates to task performance and anxiety levels in each context. Given the preliminary state of knowledge regarding emotional modulation of prefrontal inhibitory control in GAD, this study is considered exploratory in nature.

## 2. Methods

### 2.1. Participants

Between June 2023 and May 2024, patients with GAD were consecutively enrolled in the psychiatric departments of two teaching hospitals and one psychiatric clinic located in northern Taiwan. Meanwhile, HC participants were recruited from the surrounding community through local advertisements. To be eligible, participants with GAD had to fulfill the following five criteria: (1) age 18 years or older with a confirmed DSM‐5 diagnosis of GAD by a qualified psychiatrist; (2) native proficiency in Mandarin Chinese; (3) right‐hand dominance; (4) normal or corrected‐to‐normal visual acuity; and (5) intact hearing and ability to communicate verbally. HCs were required to meet the same inclusion criteria except for the GAD diagnosis. Exclusion criteria for both groups included (1) the presence of serious neurological conditions such as brain tumors or stroke; (2) comorbid major psychiatric illnesses (e.g., major depressive disorder, schizophrenia, bipolar disorder, substance use disorders, or other anxiety disorders); and (3) unstable or severe physical health conditions (e.g., acute gastrointestinal problems, cardiovascular diseases, or thyroid dysfunctions). Ethical approval was obtained from the institutional review boards of all participating sites, and written informed consent was secured from each participant prior to their involvement.

### 2.2. Nonemotional and Emotional Go/No‐Go Task

Participants performed a Go/No‐Go task to evaluate response inhibition under nonemotional and emotional conditions. They were instructed to press the key in response to target stimuli (Go trials) and withhold responses to nontargets (No‐Go trials), with a 3:1 ratio of Go to No‐Go trials. Nonemotional stimuli consisted of colored shapes, such as a blue circle as the target and a green triangle as the nontarget. Emotional stimuli involved facial expressions, where emotional faces served as targets and neutral faces as nontargets.

**Figure 1:**
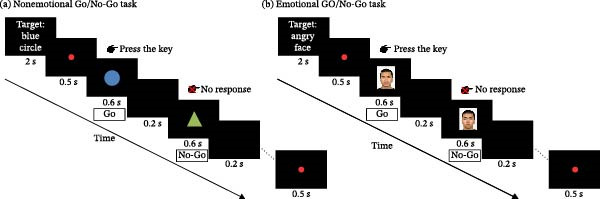
Examples of the (a) nonemotional Go/No‐Go task and (b) emotional Go/No‐Go task.

Before the formal task, participants received standardized instructions and completed a brief practice block to ensure task comprehension. The main task consisted of four blocks presented in a fixed order: two nonemotional blocks, followed by two emotional blocks. Each block began with a 2‐s cue showing the target type, followed by a 0.5‐s red fixation dot indicating the start of the block. During each trial, a stimulus was displayed for 0.6 s, followed by a 0.2‐s interstimulus interval, totaling 40 trials per block. Upon completion of each block, a 0.5‐s red dot was displayed, followed by a 30‐s rest period. No‐Go accuracy (inhibitory control), Go accuracy (sustained attention), and reaction time on correct Go trials were recorded as indicators of task performance. A schematic overview of the nonemotional and emotional Go/No‐Go tasks is presented in Figure 1.

### 2.3. Prefrontal Activity Measurement and Data Acquisition

**Figure 2:**
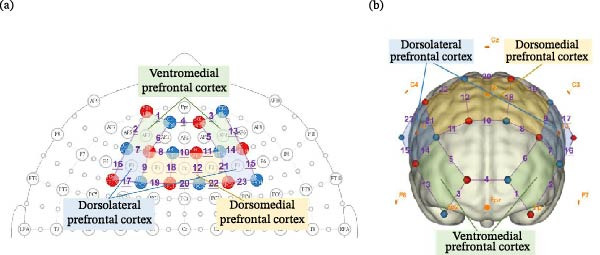
fNIRS optode configuration consisting of 8 sources (red circles), 8 detectors (blue circles), and 23 measurement channels (purple underlined numbers), covering the ventromedial, dorsomedial, and dorsolateral regions of the prefrontal cortex shown in (a) a 2D layout based on the international 10–10 system and (b) a 3D projection onto a head model.

A continuous‐wave functional near‐infrared spectroscopy (fNIRS) system (NIRSport2, NIRx Medizintechnik GmbH, Berlin, Germany) was used to monitor hemodynamic responses in the bilateral PFC during the Go/No‐Go task. The device emitted near‐infrared light at 760 and 850 nm and sampled signals at 10.17 Hz. A total of eight sources and eight detectors were arranged on the scalp according to the international 10–5 system, covering the VMPFC, DMPFC, and DLPFC on both hemispheres (see Figure 2). Channel‐to‐region correspondence was determined based on the scalp locations of source‐detector pairs relative to standard 10–5 electrode positions, following established fNIRS conventions for prefrontal mapping [21]. Each source‐detector pair was spaced ~3 cm apart. Data acquisition was conducted using Aurora software (Version 2023.9.3), and the signal quality was verified before recording.

The recordings took place in a quiet, dimly lit room. The participants were seated comfortably in front of a monitor and a single‐key keyboard. To reduce motion artifacts, the players were instructed to minimize movement throughout the recording.

### 2.4. Data Preprocessing

Preprocessing of the fNIRS data was performed using Homer3, an open‐source MATLAB‐based software package. Raw signals were first visually examined to assess signal quality, detect artifacts, and check source‐detector connectivity. Channels with poor signal quality or excessive noise were excluded from further analysis. The remaining light intensity data were then converted into optical density.

A bandpass filter between 0.001 and 0.1 Hz was applied to minimize the effects of physiological noise and slow signal drifts. Subsequently, changes in concentrations of oxygenated (ΔHbO) and deoxygenated hemoglobin were calculated using the modified Beer–Lambert Law [22]. In this study, ΔHbO was chosen as the primary metric for cortical activity as it has been shown to more reliably reflect task‐evoked cerebral blood flow changes compared to deoxygenated hemoglobin [23].

For each participant, mean ΔHbO values were computed for every channel during the nonemotional and emotional task blocks. To account for the delayed nature of the hemodynamic response [24], data analysis was restricted to the time window beginning 5 s after task onset and extending to the end of each block. To reduce interindividual variability, ΔHbO signals within each block were normalized using the mean value from the first 5 s of that same block as a reference. Finally, responses across the two emotional and two nonemotional blocks were averaged separately to generate a representative activity value for each condition.

Channels with poor signal quality were excluded prior to analysis on a participant‐by‐participant basis. In the GAD group, one channel‐participant pair in total (Channel 1, *n* = 1) was excluded. In the HC group, seven channel‐participant pairs in total (Channel 8, *n* = 2; Channel 11, *n* = 5) were excluded. All remaining channels were retained for analysis.

### 2.5. Clinical Anxiety Assessment

The state‐trait anxiety inventory (STAI) is a widely used self‐report measure designed to evaluate both transient and enduring aspects of anxiety [25]. Comprising 40 items, the STAI includes two subscales: the STAI‐state anxiety scale (STAI‐S), which captures momentary emotional states, and the STAI‐trait anxiety scale (STAI‐T), which reflects a person’s general predisposition to anxiety. Each item was rated on a 4‐point Likert scale, with subscale scores ranging from 20 to 80. Higher scores correspond to higher levels of anxiety. The STAI has demonstrated strong psychometric properties in both clinical and nonclinical populations [26]. The participants completed the STAI immediately after the tasks.

### 2.6. Statistical Analysis

All statistical analyses were conducted using SPSS (Version 29; IBM Corp., Armonk, NY, USA). Descriptive statistics were used to summarize the participant demographics, task performance, and anxiety scores. Group comparisons between individuals with GAD and HC were conducted using chi‐square tests for categorical variables and independent sample *t*‐tests for continuous variables.

To examine the effects of emotional context and group on prefrontal activity, a series of two‐way repeated‐measures ANOVAs were performed for each of the 23 fNIRS channels, with group (GAD vs. HC) as the between‐subjects factor and condition (emotional vs. nonemotional Go/No‐Go) as the within‐subjects factor. This approach allows simultaneous assessment of the group × condition interaction (i.e., the primary hypothesis of interest) along with group and condition main effects within a single factorial model. Significance was set at *p* < 0.05. To control for multiple comparisons across the 23 fNIRS channels, *p*‐values were adjusted using the Benjamini–Hochberg false discovery rate (FDR) correction method for each effect type (group × condition interaction, group main effect, and condition main effect) [27, 28]. Both uncorrected and FDR‐corrected *p*‐values are reported throughout. Effect sizes are expressed as *η*_p_^2^, with ≥0.01, ≥0.06, and ≥0.14 representing small, medium, and large effects, respectively [29].

The associations between prefrontal activity and anxiety measures, as well as between prefrontal activity and task performance, were examined separately for each group using Pearson correlation analyses. Specifically, correlations were computed between average ΔHbO in each task condition and the participants’ scores on the STAI‐S, STAI‐T, Go accuracy, No‐Go accuracy, and reaction time. To control for multiple comparisons, FDR correction was applied separately for each behavioral and anxiety measure across the 23 fNIRS channels. Both uncorrected and FDR‐corrected *p*‐values are reported alongside the correlation coefficient. Correlation coefficients with an absolute value of 0.40 or higher were considered meaningful [30].

## 3. Results

### 3.1. Participant Characteristics and Task Performance

A total of 23 patients with GAD and 42 HC were included in the study. Table 1 shows the characteristics of the participants. No significant group differences were observed in demographic variables, including age (*p* = 0.279), gender distribution (*p* = 0.178), education level (*p* = 0.523), marital status (*p* = 0.476), or employment status (*p* = 0.309). As expected, anxiety levels were significantly higher in the GAD group. The mean STAI‐S score was 47.39 in the GAD group, compared to 31.48 in the HC group (*p* < 0.001). Similarly, the GAD group reported a higher STAI‐T score, with a mean of 57.96, while the HC group had a mean of 37.50 (*p* < 0.001).

**Table 1:** Characteristics of patients with generalized anxiety disorder and healthy controls.

Characteristics	GAD (*n* = 23)	HC (*n* = 42)	GAD vs. HC (*p* value)
Age (years)	51.13 ± 14.54	57.00 ± 23.35	0.279
Gender (male/female)	5/18	16/26	0.178
Education level (≤high school/≥college)	8/14	12/30	0.523
Marriage (single or divorced/married, separated, or widowed)	7/15	16/23	0.476
Employment (full‐time or part‐time or volunteer/unemployed or homemaker)	10/12	23/16	0.309
STAI‐S score	47.39 ± 11.21	31.48 ± 8.76	<0.001
STAI‐T score	57.96 ± 10.98	37.50 ± 11.49	<0.001

*Note:* Values are means ± standard deviations.

Abbreviations: GAD, generalized anxiety disorder; HC, healthy controls; STAI‐S, state‐trait anxiety inventory‐state anxiety scale; STAI‐T, state‐trait anxiety inventory‐trait anxiety scale.

Table 2 shows the task performance by group and condition, along with the ANOVA results. For Go accuracy, both the group × condition interaction (*p* = 0.941, *η*_p_^2^ < 0.001) and group main effect were not significant (*p* = 0.450, *η*_p_^2^ = 0.010), with comparable Go accuracy between GAD and HC across conditions. A significant condition main effect was observed (*p* = 0.001, *η*_p_^2^ = 0.179), with both groups showing lower Go accuracy during the emotional task than the nonemotional task (GAD: 96.76% vs. 92.54%; HC: 95.79% vs. 91.74%).

**Table 2:** Effects of group and task condition on task performance.

Task performance	GAD (*n* = 23)	HC (*n* = 42)	Group × condition			Group			Condition		
	Mean ± SD	Mean ± SD	*F*	*p*	*η*_p_^2^	*F*	*p*	*η*_p_^2^	*F*	*p*	*η*_p_^2^
Go accuracy			0.005	0.941	<0.001	0.578	0.450	0.010	13.106	0.001^∗^	0.179
Nonemotional Go/No‐Go	96.76 ± 2.95	95.79 ± 4.74	—	—	—	—	—	—	—	—	—
Emotional Go/No‐Go	92.54 ± 4.65	91.74 ± 8.31	—	—	—	—	—	—	—	—	—
No‐Go accuracy			4.817	0.032^∗^	0.074	0.666	0.418	0.011	268.559	<0.001^∗^	0.817
Nonemotional Go/No‐Go	87.70 ± 12.30	79.49 ± 18.69	—	—	—	—	—	—	—	—	—
Emotional Go/No‐Go	47.17 ± 12.94	48.54 ± 19.68	—	—	—	—	—	—	—	—	—
Reaction time			2.985	0.089	0.047	1.736	0.193	0.028	66.855	0.001^∗^	0.527
Nonemotional Go/No‐Go	0.36 ± 0.05	0.36 ± 0.05	—	—	—	—	—	—	—	—	—
Emotional Go/No‐Go	0.45 ± 0.03	0.42 ± 0.06	—	—	—	—	—	—	—	—	—

*Note: η*_p_^2^, partial eta squared. Condition = emotional vs. nonemotional Go/No‐Go task; Group = GAD vs. HC.

^∗^ *p* < 0.05.

For No‐Go accuracy, a significant group × condition interaction was observed (*p* = 0.032, *η*_p_^2^ = 0.074). During the nonemotional task, GAD showed higher No‐Go accuracy than HC (87.70% vs. 79.49%), whereas both groups performed comparably during the emotional task (GAD: 47.17%; HC: 48.54%). The group main effect was not significant (*p* = 0.418, *η*_p_^2^ = 0.011). A significant condition main effect was observed (*p* < 0.001, *η*_p_^2^ = 0.817), with both groups showing markedly lower No‐Go accuracy during the emotional task (GAD: 87.70% vs. 47.17%; HC: 79.49% vs. 48.54%).

For reaction time, the group × condition interaction (*p* = 0.089, *η*_p_^2^ = 0.047) and the group main effect were not significant (*p* = 0.193, *η*_p_^2^ = 0.028), with both groups showing comparable reaction times across conditions. A significant condition main effect was observed (*p* < 0.001, *η*_p_^2^ = 0.527), with both groups showing longer reaction times during the emotional task than the nonemotional task (GAD: 0.36 s vs. 0.45 s; HC: 0.36 s vs. 0.42 s).

### 3.2. Prefrontal Activity Across Tasks in Both Groups

**Figure 3:**
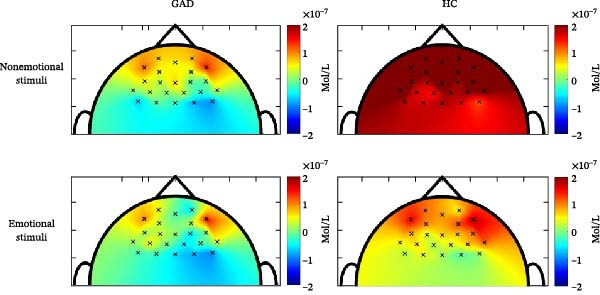
Topographical heatmaps of mean ΔHbO during nonemotional Go/No‐Go task (first row) and emotional Go/No‐Go task (second row) in the GAD group (first column) and HC group (second column).

Table 3 shows the prefrontal activity by group and condition, along with the ANOVA results. The topographical distribution of prefrontal activity across tasks in both groups is illustrated in Figure 3. A significant group × condition interaction was observed in the left DLPFC (Channel 16: *p* = 0.047, *η*_p_^2^ = 0.061; Channel 17: *p* = 0.013, *η*_p_^2^ = 0.094) and right DMPFC (Channel 22: *p* = 0.038, *η*_p_^2^ = 0.067), all reflecting medium effect sizes; however, none of these interactions survived FDR correction (FDR‐adjusted *p* = 0.288). The HC showed substantially reduced ΔHbO during the emotional relative to the nonemotional task in these channels (Channel 16: 0.23 vs. 0.05 μM; Channel 17: 0.17 vs. 0.02 μM; and Channel 22: 0.16 vs −0.02 μM), whereas the GAD group showed a comparatively flat and unmodulated profile (Channel 16: 0.01 vs. 0.01 μM; Channel 17: −0.07 vs. −0.03 μM; and Channel 22: −0.08 vs. −0.08 μM). An uncorrected significant group main effect of medium magnitude was observed across 11 channels spanning the right VMPFC, bilateral DMPFC, and bilateral DLPFC (Channels 4, 5, 11, 12, 14, 17, 19, 20, 21, 22, and 23: *p* = 0.005–0.039, *η*_p_^2^ = 0.066–0.130), with none surviving FDR correction (FDR‐adjusted *p* = 0.052–0.082). Across all 11 channels, GAD showed a lower average ΔHbO than HC across both conditions. A significant condition main effect was observed in seven channels covering right VMPFC and right DMPFC (Channels 3, 4, 10, 11, 12, 20, and 22: *p* = 0.001–0.042, FDR‐adjusted *p* = 0.023–0.138, *η*_p_^2^ = 0.064–0.159), with Channels 3, 4, and 12 remaining significant after FDR correction.

**Table 3:** Effects of group and task condition on prefrontal activity.

Channel	GAD (*n* = 23), mean ± SD (μM)		HC (*n* = 42), mean ± SD (μM)		Group × condition			Group			Condition		
	Nonemotional	Emotional	Nonemotional	Emotional	*p*	*p*_adj	*η*_p_^2^	*p*	*p*_adj	*η*_p_^2^	*p*	*p*_adj	*η*_p_^2^
Ch 1	0.07 ± 0.34	0.04 ± 0.17	0.23 ± 0.33	0.10 ± 0.32	0.254	0.377	0.021	0.099	0.134	0.043	0.097	0.173	0.044
Ch 2	0.11 ± 0.22	0.10 ± 0.20	0.23 ± 0.40	0.15 ± 0.31	0.538	0.552	0.006	0.148	0.162	0.033	0.417	0.417	0.010
Ch 3	0.07 ± 0.45	−0.06 ± 0.37	0.25 ± 0.37	0.05 ± 0.41	0.552	0.552	0.006	0.101	0.134	0.042	0.004^∗^	0.038^∗^	0.127
Ch 4	0.05 ± 0.27	−0.03 ± 0.20	0.21 ± 0.26	0.09 ± 0.31	0.472	0.517	0.008	0.028^∗^	0.071	0.074	0.005^∗^	0.038^∗^	0.120
Ch 5	0.04 ± 0.35	0.03 ± 0.25	0.21 ± 0.25	0.12 ± 0.30	0.353	0.406	0.014	0.039^∗^	0.082	0.066	0.222	0.281	0.024
Ch 6	0.05 ± 0.19	0.02 ± 0.20	0.21 ± 0.30	0.10 ± 0.30	0.214	0.352	0.024	0.056	0.106	0.057	0.064	0.138	0.054
Ch 7	0.04 ± 0.26	0.01 ± 0.21	0.20 ± 0.39	0.08 ± 0.31	0.311	0.377	0.016	0.105	0.134	0.041	0.066	0.138	0.053
Ch 8	0.02 ± 0.26	0.03 ± 0.17	0.17 ± 0.31	0.03 ± 0.26	0.064	0.287	0.055	0.195	0.195	0.027	0.113	0.173	0.041
Ch 9	0.03 ± 0.21	0.05 ± 0.24	0.16 ± 0.22	0.06 ± 0.26	0.115	0.294	0.039	0.146	0.162	0.033	0.325	0.340	0.015
Ch 10	0.07 ± 0.26	0.01 ± 0.25	0.25 ± 0.36	0.07 ± 0.46	0.171	0.326	0.030	0.157	0.164	0.032	0.010^∗^	0.058	0.100
Ch 11	−0.02 ± 0.25	−0.06 ± 0.23	0.18 ± 0.20	0.07 ± 0.28	0.310	0.377	0.018	0.005^∗^	0.052	0.130	0.022^∗^	0.084	0.087
Ch 12	0.03 ± 0.20	−0.05 ± 0.21	0.18 ± 0.21	0.04 ± 0.27	0.306	0.377	0.017	0.014^∗^	0.064	0.092	0.001^∗^	0.023^∗^	0.159
Ch 13	0.14 ± 0.28	0.15 ± 0.21	0.29 ± 0.28	0.18 ± 0.28	0.174	0.326	0.029	0.097	0.134	0.043	0.244	0.281	0.021
Ch 14	−0.02 ± 0.33	−0.01 ± 0.24	0.23 ± 0.29	0.10 ± 0.30	0.092	0.287	0.045	0.008^∗^	0.052	0.106	0.107	0.173	0.041
Ch 15	0.03 ± 0.31	0.04 ± 0.26	0.22 ± 0.32	0.12 ± 0.36	0.183	0.326	0.028	0.072	0.118	0.051	0.304	0.333	0.017
Ch 16	0.01 ± 0.19	0.01 ± 0.19	0.23 ± 0.45	0.05 ± 0.27	0.047^∗^	0.287	0.061	0.060	0.106	0.055	0.060	0.138	0.055
Ch 17	−0.07 ± 0.13	−0.03 ± 0.30	0.17 ± 0.26	0.02 ± 0.26	0.013^∗^	0.287	0.094	0.005^∗^	0.052	0.117	0.188	0.254	0.027
Ch 18	0.00 ± 0.27	−0.00 ± 0.22	0.14 ± 0.20	0.02 ± 0.29	0.095	0.287	0.044	0.135	0.162	0.035	0.064	0.138	0.053
Ch 19	−0.02 ± 0.21	−0.03 ± 0.24	0.16 ± 0.24	0.04 ± 0.30	0.184	0.326	0.028	0.023^∗^	0.066	0.079	0.107	0.173	0.041
Ch 20	−0.04 ± 0.24	−0.07 ± 0.24	0.18 ± 0.24	0.02 ± 0.32	0.100	0.287	0.042	0.009^∗^	0.052	0.104	0.019^∗^	0.084	0.085
Ch 21	−0.03 ± 0.44	−0.02 ± 0.29	0.20 ± 0.21	0.07 ± 0.30	0.082	0.287	0.047	0.023^∗^	0.066	0.080	0.164	0.236	0.031
Ch 22	−0.08 ± 0.34	−0.08 ± 0.30	0.16 ± 0.30	−0.02 ± 0.32	0.038^∗^	0.287	0.067	0.031^∗^	0.071	0.072	0.042^∗^	0.138	0.064
Ch 23	−0.09 ± 0.47	−0.10 ± 0.27	0.12 ± 0.22	0.01 ± 0.30	0.292	0.377	0.018	0.018^∗^	0.066	0.085	0.241	0.281	0.022

*Note: η*_p_^2^, partial eta squared. Condition = emotional vs. nonemotional Go/No‐Go task; Group = GAD vs. HC.

^∗^ *p* < 0.05.

### 3.3. Correlation of Task Performance, Anxiety, and Cortical Activity Within Each Group

**Figure 4:**
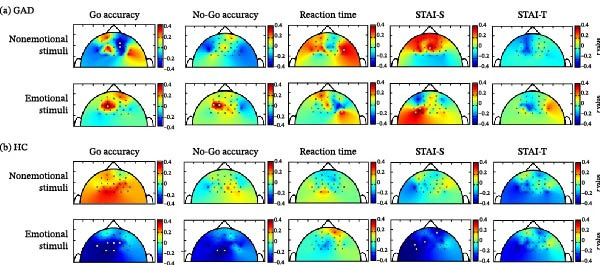
Correlations between prefrontal ΔHbO concentration and task performance as well as anxiety measures in (a) the GAD group and (b) healthy controls during nonemotional (first row) and emotional (second row) Go/No‐Go tasks. White crosses denote channels with notable correlations (|*r*| ≥ 0.4). Abbreviations: GAD, generalized anxiety disorder; HC, healthy controls; STAI‐S, state‐trait anxiety inventory‐state anxiety scale; STAI‐T, state‐trait anxiety inventory‐trait anxiety scale.

Figure 4 presents the correlations between prefrontal activity and both task performance and anxiety measures within each group. Association patterns differed by task type and by group. In the GAD group, during the nonemotional task, decreased activity in the right VMPFC (Channel 5: *r* = −0.52, *p* = 0.018, FDR‐adjusted *p* = 0.253) and right DMPFC (Channel 11: *r* = −0.51, *p* = 0.022, FDR‐adjusted *p* = 0.253) was notably associated with higher Go accuracy. No notable associations were found between prefrontal activity and No‐Go accuracy during the nonemotional task. Increased activity in the right DLPFC was notably associated with longer reaction time (Channel 15: *r* = 0.41, *p* = 0.071, FDR‐adjusted *p* = 0.836). Greater activity in the DMPFC correlated with higher STAI‐S scores (Channel 10: *r* = 0.45, *p* = 0.033, FDR‐adjusted *p* = 0.613). On the other hand, under emotional task, greater activity in the left DMPFC (Channel 8) was notably associated with both higher Go accuracy (*r* = 0.54, *p* = 0.014, FDR‐adjusted *p* = 0.322) and higher No‐Go accuracy (*r* = 0.57, uncorrected *p* = 0.009, FDR‐adjusted *p* = 0.207). Activity in the left DLPFC (Channel 7) showed a notable association specifically with higher No‐Go accuracy (*r* = 0.47, *p* = 0.035, FDR‐adjusted *p* = 0.403) but not with Go accuracy (*r* = 0.29, *p* = 0.209). Increased activity in the right DLPFC was correlated with longer reaction time (Channel 23: *r* = 0.45, *p* = 0.049, FDR‐adjusted *p* = 0.974). Additionally, the left DLPFC showed a notable positive correlation with STAI‐S scores (Channels 9 and 17: *r* = 0.41–0.43, *p* = 0.039–0.051, FDR‐adjusted *p* = 0.586). No notable correlations were found between prefrontal activity and STAI‐T scores during either task in the GAD group.

In contrast, among HC, notable correlations emerged only during the emotional task and were consistently negative. Specifically, widespread negative correlations between prefrontal activity and Go accuracy were observed in the bilateral DMPFC and left DLPFC (Channels 8, 10, 11, 16, 19, and 20: *r* = −0.40 to −0.48, *p* = 0.001–0.009, FDR‐adjusted *p* = 0.023–0.030), with the DMPFC also showing negative associations with No‐Go accuracy (Channels 19 and 20: *r* = −0.41 to −0.50, *p* = 0.001–0.008, FDR‐adjusted *p* = 0.023–0.092). Decreased activity in the left DLPFC (Channels 16 and 17: *r* = −0.43 to −0.48, *p* = 0.001–0.005, FDR‐adjusted *p* = 0.023–0.035), left DMPFC (Channel 8: *r* = −0.46, *p* = 0.003, FDR‐adjusted *p* = 0.035), and right VMPFC (Channel 3: *r* = −0.42, *p* = 0.006, FDR‐adjusted *p* = 0.035) was related to higher STAI‐S scores. No notable correlations were observed between prefrontal activity and reaction time or STAI‐T scores under either task.

## 4. Discussion

This study investigated the impact of emotion on prefrontal activity during inhibitory control in patients with GAD compared to HC using a Go/No‐Go task with nonemotional and emotional stimuli. The primary findings converged across behavioral, neural, and brain–behavior levels on a pattern of differential emotional modulation between groups. Behaviorally, a significant group × condition interaction was observed for No‐Go accuracy. Specifically, GAD showed a larger decline in No‐Go accuracy from the nonemotional to the emotional task than HC, indicating that GAD was more susceptible to emotional interference on response inhibition. At the neural level, the medium‐sized group × condition interaction reached significance (uncorrected) in the left DLPFC and right DMPFC. HC showed reduced activation in these prefrontal regions from the nonemotional to the emotional task, whereas GAD showed negligible change, reflecting absent context‐sensitive prefrontal modulation. At the brain–behavior level, the left DLPFC further emerged as a key region. In GAD, its activation during the emotional task was selectively associated with higher No‐Go accuracy but not Go accuracy, converging with the neural group × condition interaction to implicate the left DLPFC as the primary locus of emotion‐sensitive inhibitory control deficits in GAD. In contrast, HC showed the opposing pattern, with lower left DLPFC and DMPFC activation associated with higher accuracy and higher state anxiety, indicating efficient prefrontal regulation.

The Go/No‐Go task used in this study primarily assesses proactive inhibition, the ability to engage top‐down control in anticipation of infrequent No‐Go stimuli [31], across both nonemotional and emotional contexts. The significant group × condition interaction for No‐Go accuracy reveals that emotional stimuli disproportionately disrupted response inhibition in GAD relative to HC. In the nonemotional task, GAD showed higher No‐Go accuracy than HC (87.70% vs. 79.49%), suggesting intact proactive inhibitory engagement in the absence of emotional interference. However, under the emotional task, both groups converged to similarly impaired levels (GAD: 47.17% and HC: 48.54%), reflecting a decline of 40.5 percentage points in GAD compared to 30.9 percentage points in HC. This pattern indicates that the emotional context specifically eroded GAD’s capacity for proactive response inhibition, effectively eliminating their nonemotional advantage in No‐Go performance. No group × condition interaction was found for Go accuracy, where both groups showed comparable declines (GAD: 96.76% vs. 92.54%; HC: 95.79% vs. 91.74%), or for reaction time, where both groups showed numerically longer responses under emotional conditions (GAD: 0.36 vs. 0.45 s; HC: 0.36 vs. 0.42 s) but without significant group differences. This suggests that emotional interference in GAD selectively disrupted response inhibition rather than sustained attention or overall processing speed. These findings suggest a specific breakdown in proactive inhibitory control in GAD when emotional stimuli compete for executive resources, consistent with the evidence of reduced proactive engagement in GAD in affective paradigms [32].

Our fNIRS findings reveal distinct patterns of context‐dependent prefrontal modulation between groups, most directly captured by the group × condition interaction, with medium effect sizes observed in the left DLPFC and right DMPFC. In HC, ΔHbO decreased substantially from the nonemotional to the emotional task in these regions, suggesting context‐sensitive downregulation of prefrontal resources. In contrast, GAD showed negligible change across conditions in the same channels, consistent with a pattern of reduced dynamic prefrontal modulation. This pattern of prefrontal inflexibility in GAD aligns with prior neuroimaging evidence showing consistently low prefrontal activity across emotional and nonemotional conditions in GAD, reflecting impaired top‐down control mechanisms [11, 33]. This may arise from attentional bias, where chronic internal anxiety signals pervasively preload their attentional resources, thereby diminishing the capacity for dynamic prefrontal modulation [34, 35]. Consequently, their prefrontal system operates at a lower, less flexible capacity, unable to differentiate its response effectively between emotional and nonemotional demands. This preliminary pattern of prefrontal inflexibility aligns with previous electrophysiological evidence showing impaired response inhibition and reduced DLPFC activity when individuals with GAD process emotional stimuli [36] and is further supported by the selective No‐Go accuracy impairment observed in the present study.

Beyond the interaction pattern, the group main effect revealed consistently lower ΔHbO in GAD relative to HC across both task conditions, with medium‐to‐large effect sizes spanning 11 channels in the DMPFC and DLPFC. The largest differences were observed in the right DMPFC and left DLPFC, suggesting that GAD exhibited broadly reduced overall prefrontal engagement regardless of the task condition. The right DMPFC and left DLPFC, regions critical for self‐monitoring and top‐down suppression, both showed GAD activation close to or below baseline, suggesting chronic underengagement of these regulatory regions. Despite this overall hypoactivity, accuracy was broadly comparable between groups in the nonemotional task; however, the underlying mechanisms of prefrontal engagement appear divergent. In HC, the pattern of overall higher activation combined with context‐dependent reduction under emotional load reflects flexible resource allocation, deploying greater prefrontal engagement when needed and downregulating under conditions of greater automaticity. In contrast, the flat and unmodulated prefrontal response observed in GAD, already at lower absolute levels across both conditions, suggests a failure to engage top‐down control when processing emotionally salient information.

Distinct patterns of brain–behavior–anxiety relationships emerged between the GAD and HC groups, reflecting divergent neural strategies for emotional and cognitive regulation. In the GAD group, the direction of prefrontal behavior associations shifted between conditions. During the nonemotional task, lower right VMPFC and right DMPFC activation was associated with higher Go accuracy, suggesting that reduced right medial prefrontal engagement may be sufficient for sustained attention in the absence of emotional interference. Under the emotional task, greater left DMPFC activation was associated with both higher Go and No‐Go accuracy, a pattern distinct from the right medial prefrontal associations observed during the nonemotional task. This shift suggests that the introduction of emotional interference recruited the left DMPFC as an additional compensatory resource, with greater engagement of this region specifically linked to better maintenance of both sustained attention and response inhibition performance. Critically, left DLPFC activation showed a specific association with higher No‐Go accuracy but not Go accuracy, suggesting that left DLPFC recruitment in GAD may be specifically associated with response inhibition rather than general performance under emotional load. Greater right DLPFC activation was independently associated with longer reaction times, indicating that prefrontal engagement in GAD extended beyond the left‐lateralized accuracy‐linked regions. Furthermore, greater left DLPFC and DMPFC activation was positively associated with state anxiety, such that individuals with GAD who reported higher momentary anxiety showed greater left dorsal prefrontal engagement, suggesting that transient anxious arousal may co‐occur with effortful recruitment of left‐lateralized emotion regulation and inhibitory control regions. The absence of notable associations with trait anxiety across both task conditions further indicates that these prefrontal responses are more sensitive to fluctuating emotional states than to stable dispositional characteristics.

In contrast, the HC group exhibited a strikingly different and more efficient brain–behavior–anxiety profile, with all notable associations emerging exclusively during the emotional task and in the opposite direction to GAD. For task accuracy, lower DMPFC and left DLPFC activation was associated with higher Go accuracy, and lower DMPFC activation was associated with higher No‐Go accuracy, indicating that HC individuals who performed better actually recruited fewer prefrontal resources. This opposing direction constitutes a double dissociation where GAD required more prefrontal engagement to achieve better inhibitory performance, whereas HC achieved better performance with less. For state anxiety, lower right VMPFC, left DMPFC, and left DLPFC activation were associated with higher STAI‐S scores, indicating that HC individuals with higher momentary anxiety paradoxically showed lower prefrontal activation. Rather than reflecting emotional dysregulation, this may suggest that within the normal range, elevated state anxiety enhanced attentional engagement without necessitating additional left dorsal prefrontal recruitment, in contrast to GAD, where higher momentary anxiety co‐occurred with greater left dorsal prefrontal effort. No notable associations between prefrontal activity and either accuracy or anxiety were observed in HC during the nonemotional task, underscoring the context specificity of these effects and the efficiency with which HC adapted prefrontal resources selectively to emotional demands.

Several limitations should be acknowledged. First, the cross‐sectional design precludes conclusions about causal relationships between anxiety, brain activity, and task performance. Second, the relatively small clinical sample size, particularly in the GAD group, limits the statistical power and generalizability. Given the exploratory nature of this study, channels with medium‐to‐large effect sizes (*η*_p_^2^ ≥ 0.06) and notable correlations (|*r*| ≥ 0.40) are interpreted as reflecting meaningful effects and associations, respectively, even when FDR‐corrected *p*‐values do not reach significance, consistent with the view that limited statistical power rather than a true null result explains the failure to survive correction. Future confirmatory studies with larger samples are warranted. Third, the medication status was not systematically recorded in the current study. Psychotropic medications, including selective serotonin reuptake inhibitors and benzodiazepines commonly prescribed for GAD, may have influenced prefrontal hemodynamic responses measured by fNIRS. Fourth, the use of photo‐based emotional stimuli may not capture the complexity of real‐life emotional experiences or clinical triggers for GAD. Fifth, the fixed sequence of task blocks (nonemotional preceding emotional) may have introduced order effects, potentially confounding the interpretation of emotional modulation. Lastly, the spatial resolution of fNIRS restricts conclusions about deeper or broader neural networks involved in emotional regulation.

## 5. Conclusions

This study provides preliminary evidence that emotional context differentially influences prefrontal activity and inhibitory performance in GAD relative to that in HC. GAD showed a disproportionate decline in No‐Go accuracy under emotional conditions and a pattern of absent context‐dependent left DLPFC and right DMPFC modulation alongside broadly lower overall prefrontal activation, with brain–behavior associations suggesting compensatory left‐lateralized dorsal prefrontal recruitment to sustain inhibitory performance under emotional load. In contrast, HC showed an opposing pattern in which lower DMPFC and left DLPFC activation was associated with better performance and higher state anxiety during the emotional task, consistent with efficient and adaptive prefrontal regulation. These preliminary findings contribute to understanding of the brain–behavior–anxiety interaction in GAD, suggesting reduced prefrontal flexibility and effortful compensatory recruitment as candidate neural features of emotion‐cognition dysregulation. Replication in larger clinical samples is needed to confirm these patterns, and the results overall underscore the role of the left DLPFC and DMPFC as key regions integrating emotional and cognitive processes under conditions of anxiety.

## Funding

This work was supported by the National Science and Technology Council, R.O.C. (Grants NSTC 112‐2321‐B‐006‐014 and NSTC 113‐2321‐B‐006‐015).

## Conflicts of Interest

The authors declare no conflicts of interest.

## Data Availability

The data that support the findings of this study are available upon request from the corresponding author. The data are not publicly available due to privacy or ethical restrictions.
